# Editorial: Errors and mistakes in human-robot interactions

**DOI:** 10.3389/frobt.2026.1813746

**Published:** 2026-04-15

**Authors:** Manuel Giuliani, Leimin Tian, Maha Salem, Nicole Mirnig

**Affiliations:** 1 Faculty of Electrical Engineering, Kempten University of Applied Sciences, Kempten, Germany; 2 CSIRO Robotics, Melbourne, VIC, Australia; 3 WhatsApp/Meta, London, United Kingdom; 4 Center for Human-Computer Interaction, University of Salzburg, Salzburg, Austria

**Keywords:** errors in human-robot interaction, failure taxonomy for robot speech interfaces, impact of robot failures on social dynamics in HRI, robot failure recovery, robot learning from imperfect human demonstrations

Robots are often showcased as precise machines that seamlessly collaborate with humans. However, reality diverges significantly, as robots encounter failures that lead to suboptimum outcomes, misunderstandings and socially awkward situations. Conversely, human-induced errors in these interactions often go undetected by robots, amplifying the complexity of the interaction dynamics on top of the uncertainties in the environment and interaction contexts.

In human-robot interaction (HRI) research, errors–wrong actions that are made due to the lack of knowledge–and mistakes–actions that turn out to be wrong–are commonly viewed as impediments to achieving flawless collaboration. Scholars and practitioners aspire to meticulously control variables, creating environments with predictable storylines and outcomes. Nevertheless, the controlled setting of a laboratory rarely mirrors the unpredictable nature of real-world scenarios, contributing to a notable disparity between expectations and actual experiences. The ability of robots to navigate erroneous situations is paramount to the sustained success of HRI. These imperfections are also perfect learning opportunities for robots to continuously adapt to the ever-shifting complexity and dynamics in real-world HRI.

This Research Topic contains research that addresses the gap between anticipated perfection and the inherent uncertainties in a diverse range of real-world applications. The papers presented here shine light on a variety of aspects ranging from novel technical approaches to repair failures in HRI, to user studies that aim to understand social dimensions of errors in HRI.

In “Working with Troubles and Failures in Conversation Between Humans and Robots: Workshop Report”, Förster et al. give a summary of the first session of an ongoing workshop series addressing topics very close to this Research Topic. The workshop brought together an interdisciplinary group, including roboticists, HRI researchers, dialogue system experts, conversation analysts, cognitive scientists, and linguists with the aim to systematically examine miscommunication in human-robot interactions. The report maps out the “failure landscape” in robotic speech interfaces, and explores the feasibility of creating a standardized benchmark scenario for testing future systems. It outlines the key failures identified and the progress made towards defining such a benchmark, marking a foundational step for advancing robust human-robot communication.

In their article “Helping or Watching it Happen: How Participants Respond to Robot Failures in a Turn-Taking Game”, Stedtler et al. explore in a study how people react in real time to robot malfunctions, such as motion interruptions or failed object manipulation, during Tic-Tac-Toe games with the humanoid robot Epi. Their focus is on how social roles, responsibilities, and agency are negotiated. The study findings reveal that humans actively support robots through physical repairs, emotional care, and interpretive help, often shouldering the burden of maintaining interactional continuity and redefining agency in the process.

The study “Should Robots Display What They Hear? Mishearing as a Practical Accomplishment” by Rudaz and Licoppe explores how publicly displaying a robot’s automatic speech recognition (ASR) results influences the emergence and resolution of miscommunications in human–robot interaction. Through micro-analytic examination, the research reveals that participants often reinterpreted a robot’s action as problematic only after reading the ASR transcript, highlighting how this informational ecology shapes perceptions of interactional failures. The findings suggest that “errors” and “mistakes” are not pre-defined technical or social categories but emerge dynamically through interaction, fundamentally altering how mutual understanding is achieved between humans and robots.

In “Exploring the Effect of Automation Failure on the Human’s Trustworthiness in Human-Agent Teamwork”, Jorge et al. go beyond physical robots to study humans interacting with automated systems. The authors examine how automation failures in human-automation teams impact both human trust in automation and human trustworthiness—the reliability of human behavior towards the automated system. Using a 2 × 2 mixed-design experiment in a simulated “moving-out” task, researchers measured participants’ trust, trustworthiness, and liking of the automation, both subjectively and objectively. The results reveal that automation failures reduce human trust, trustworthiness, and liking toward the system, highlighting the fragility of these dynamics in collaborative settings. Understanding these effects can help improve the design and functionality of human-automation teamwork, fostering more resilient and effective partnerships.

In “Error Recovery in Wearable Robotic Co-Grasping: the Role of Human-led Correction,” Chang et al. introduce Co-Grasping, a novel strategy for wearable robotic devices that enables users to dynamically allocate control between their own body power and robotic assistance during grasping tasks, particularly when errors occur. Experimental results demonstrate that while recoverable robot errors initially reduce trust and increase physical engagement, trust rebounds and behavior normalizes once the robot regains reliability. Their findings suggest that allowing users to intervene and correct faults not only prevents task failure but also helps restore trust in the system. This approach highlights the potential for adaptive human-robot collaboration, even in imperfect automated systems, by empowering users to actively manage role allocation.

In their study “Interactively Learning Behavior Trees From Imperfect Human Demonstrations”, Scherf et al. investigate robot learning from imperfect human demonstrations. This study introduces a novel interactive task-learning framework that enables robots to learn Behavior Trees (BTs) from just a few human demonstrations recorded as RGB-D video streams, eliminating the need for step-by-step instruction or full retraining. By automatically extracting pre- and post-conditions from visual features and using a Backchaining approach, the framework builds reactive BTs. A user study identifies three common failure cases when learning from imperfect demonstrations. To address these failures, the system detects issues during execution and allows users to interactively refine the BT through a web interface, adapting the tree without restarting the teaching process. The approach was evaluated in a robotic trash disposal task with 20 participants, demonstrating its ability to learn effectively from minimal demonstrations and resolve failures in real time, enhancing adaptability in human-robot collaboration.

This Research Topic provides insights on defining, detecting, preventing, and recovering from errors and mistakes in HRI, informing future research and development of adaptive and reliable systems.

